# Supplementary material for: A Characterization and an Evolutionary and a Pathogenicity Analysis of Reassortment H3N2 Avian Influenza Virus in South China in 2019–2020
Source: Viruses. 2022 Nov 21;14(11):2574. doi: 10.3390/v14112574 (PMC9692712; doi:10.3390/v14112574)
Supplement: Supplementary file 1 [file viruses-14-02574-s001.zip › Supplementary table S1.pdf]

Table S1. H3N2 strains isolated from 2019 to 2020.

Name	Date	Host	Location	Isolate ID
A/Chicken/Guangdong/G152/2019/H3N2	2019-03-04	Chicken	Guangdong	EPI_ISL_13732930
A/Chicken/Guangdong/G155/2019/H3N2	2019-03-04	Chicken	Guangdong	EPI_ISL_13732931
A/Environment/Guangdong/G188/2019/H3N2	2019-03-06	Environment	Guangdong	EPI_ISL_13732933
A/Goose/Guangdong/G630/2019/H3N2	2019-08-21	Goose	Guangdong	EPI_ISL_13732934
A/Duck/Guangdong/H34/2020/H3N2	2020-01-07	Duck	Guangdong	EPI_ISL_13732935
A/Duck/Guangdong/H140/2020/H3N2	2020-06-04	Duck	Guangdong	EPI_ISL_13732936
A/Duck/Guangdong/H144/2020/H3N2	2020-06-04	Duck	Guangdong	EPI_ISL_13732937
A/Duck/Guangxi/H151/2020/H3N2	2020-06-09	Duck	Guangxi	EPI_ISL_13734227
A/Chicken/Sichuan/H157/2020/H3N2	2020-06-09	Chicken	Sichuan	EPI_ISL_13734228
A/Chicken/Guangdong/H159/2020/H3N2	2020-06-09	Chicken	Guangdong	EPI_ISL_13734229

All isolates except A/Environment/Guangdong/G188/2019/H3N2 were isolated from Non-vaccinated and asymptomatic avian.
