# Supplementary material for: A Characterization and an Evolutionary and a Pathogenicity Analysis of Reassortment H3N2 Avian Influenza Virus in South China in 2019–2020
Source: Viruses. 2022 Nov 21;14(11):2574. doi: 10.3390/v14112574 (PMC9692712; doi:10.3390/v14112574)
Supplement: Supplementary file 1 [file viruses-14-02574-s001.zip › Supplementary table S2.pdf]

Table S2a. Nucleotide and protein homology of HA gene of ten strains.

Strain name	Sequence identities with some strains (nt/aa%)								
	34_2020	140_2020	144_2020	151_2020	157_2020	159_2020	G152_2019	G155_2019	G188_2019
34_2020	-	99.24/99.12	99.24/99.29	99.29/99.29	98.59/99.12	99.29/99.29	92.83/96.83	92.06/96.65	95.06/97.71
140_2020	99.24/99.12	-	99.88/99.82	99.94/99.82	99.12/99.29	99.82/99.82	92.53/95.94	91.53/95.77	94.53/96.83
144_2020	99.24/99.29	99.88/99.82	-	99.94/100.00	99.12/99.47	99.82/100.00	92.53/96.12	91.53/95.94	94.53/97.00
151_2020	99.29/99.29	99.94/99.82	99.94/100.00	-	99.18/99.47	99.88/100.00	92.59/96.12	91.59/95.94	94.59/97.00
157_2020	98.59/99.12	99.12/99.29	99.12/99.47	99.18/99.47	-	99.06/99.47	92.71/95.94	91.71/95.77	94.94/96.83
159_2020	99.29/99.29	99.82/99.82	99.82/100.00	99.88/100.00	99.06/99.47	-	92.59/96.12	91.59/95.94	94.59/97.00
G152_2019	92.83/96.83	92.53/95.94	92.53/96.12	92.59/96.12	92.71/95.94	92.59/96.12	-	94.06/97.71	92.12/96.47
G155_2019	92.06/96.65	91.53/95.77	91.53/95.94	91.59/95.94	91.71/95.77	91.59/95.94	94.06/97.71	-	92.24/96.65
G188_2019	95.06/97.71	94.53/96.83	94.53/97.00	94.59/97.00	94.94/96.83	94.59/97.00	92.12/96.47	92.24/96.65	-
G630_2019	95.65/98.94	95.24/98.06	95.36/98.24	95.30/98.24	95.06/98.06	95.30/98.24	91.36/96.65	91.48/96.47	94.12/98.06

Table S2b. Nucleotide and protein homology of NA gene of ten strains.

Strain name	Sequence identities with some strains (nt/aa%)								
	159_2020	157_2020	151_2020	144_2020	140_2020	34_2020	G152_2019	G155_2019	G188_2019
159_2020	-	100.00/100.00	99.43/98.72	99.93/100.00	100.00/100.00	96.03/97.23	91.42/95.53	92.34/95.74	95.04/97.23
157_2020	100.00/100.00	-	99.43/98.72	99.93/100.00	100.00/100.00	96.03/97.23	91.42/95.53	92.34/95.74	95.04/97.23
151_2020	99.43/98.72	99.43/98.72	-	99.36/98.72	99.43/98.72	95.60/95.96	90.99/94.89	92.06/94.89	94.61/95.96
144_2020	99.93/100.00	99.93/100.00	99.36/98.72	-	99.93/100.00	95.96/97.23	91.49/95.53	92.41/95.74	95.11/97.23
140_2020	100.00/100.00	100.00/100.00	99.43/98.72	99.93/100.00	-	96.03/97.23	91.42/95.53	92.34/95.74	95.04/97.23
34_2020	96.03/97.23	96.03/97.23	95.60/95.96	95.96/97.23	96.03/97.23	-	91.28/95.74	92.62/96.38	95.60/97.66
G152_2019	91.42/95.53	91.42/95.53	90.99/94.89	91.49/95.53	91.42/95.53	91.28/95.74	-	92.77/95.53	92.55/96.17
G155_2019	92.34/95.74	92.34/95.74	92.06/94.89	92.41/95.74	92.34/95.74	92.62/96.38	92.77/95.53	-	93.69/96.81
G188_2019	95.04/97.23	95.04/97.23	94.61/95.96	95.11/97.23	95.04/97.23	95.60/97.66	92.55/96.17	93.69/96.81	-
G630_2019	93.83/97.23	93.83/97.23	93.55/96.38	93.90/97.23	93.83/97.23	94.40/97.45	91.35/96.17	91.99/96.38	95.96/97.45

Table S2c. Nucleotide and protein homology of PB2 gene of ten strains.

Strain name	Sequence identities with some strains (nt/aa%)								
	G152_2019	G155_2019	G188_2019	G630_2019	H159_2020	34_2020	140_2020	144_2020	151_2020
G152_2019	-	92.98/98.68	93.51/98.82	91.05/98.55	92.94/97.24	93.77/99.08	93.68/98.68	92.68/95.79	88.20/96.84
G155_2019	92.98/98.68	-	93.95/99.34	90.26/98.82	93.42/97.37	94.78/99.47	94.34/98.82	93.07/95.79	88.29/97.11
G188_2019	93.51/98.82	93.95/99.34	-	90.61/99.21	94.91/97.63	96.14/99.61	95.66/99.08	94.69/95.92	88.55/97.37
G630_2019	91.05/98.55	90.26/98.82	90.61/99.21	-	89.87/97.11	90.57/99.08	90.53/98.55	89.30/95.39	87.32/97.11
H159_2020	92.94/97.24	93.42/97.37	94.91/97.63	89.87/97.11	-	97.54/97.89	99.08/98.55	97.41/95.13	90.92/97.50
34_2020	93.77/99.08	94.78/99.47	96.14/99.61	90.57/99.08	97.54/97.89	-	98.29/99.34	97.32/96.18	89.82/97.37
140_2020	93.68/98.68	94.34/98.82	95.66/99.08	90.53/98.55	99.08/98.55	98.29/99.34	-	98.16/96.58	90.66/97.24
144_2020	92.68/95.79	93.07/95.79	94.69/95.92	89.30/95.39	97.41/95.13	97.32/96.18	98.16/96.58	-	89.25/94.08
151_2020	88.20/96.84	88.29/97.11	88.55/97.37	87.32/97.11	90.92/97.50	89.82/97.37	90.66/97.24	89.25/94.08	-
157_2020	90.22/97.89	90.61/98.16	90.88/98.42	88.29/98.16	92.32/97.11	92.41/98.42	92.98/98.55	92.41/96.71	92.76/96.84

Table S2d. Nucleotide and protein homology of PB1 gene of ten strains.

Strain name	Sequence identities with some strains (nt/aa%)								
	H159_2020	34_2020	140_2020	144_2020	151_2020	157_2020	G155_2019	G188_2019	G630_2019
H159_2020	-	99.03/99.34	99.87/99.74	99.96/99.87	90.38/98.02	99.91/99.87	92.84/98.81	89.81/97.89	89.20/98.16
34_2020	99.03/99.34	-	99.08/99.34	99.08/99.47	90.73/98.68	99.03/99.47	92.97/99.21	89.90/98.55	89.33/98.42
140_2020	99.87/99.74	99.08/99.34	-	99.91/99.87	90.43/98.02	99.87/99.87	92.84/98.81	89.77/97.89	89.24/98.16
144_2020	99.96/99.87	99.08/99.47	99.91/99.87	-	90.43/98.16	99.96/100.00	92.89/98.95	89.86/98.02	89.24/98.29
151_2020	90.38/98.02	90.73/98.68	90.43/98.02	90.43/98.16	-	90.38/98.16	90.56/98.16	90.69/98.29	90.47/98.29
157_2020	99.91/99.87	99.03/99.47	99.87/99.87	99.96/100.00	90.38/98.16	-	92.93/98.95	89.81/98.02	89.20/98.29
G155_2019	92.84/98.81	92.97/99.21	92.84/98.81	92.89/98.95	90.56/98.16	92.93/98.95	-	90.51/98.02	89.15/98.02
G188_2019	89.81/97.89	89.90/98.55	89.77/97.89	89.86/98.02	90.69/98.29	89.81/98.02	90.51/98.02	-	94.20/98.42
G630_2019	89.20/98.16	89.33/98.42	89.24/98.16	89.24/98.29	90.47/98.29	89.20/98.29	89.15/98.02	94.20/98.42	-
G152_2019	97.01/98.55	97.01/99.21	96.97/98.55	97.06/98.68	90.34/98.55	97.10/98.68	93.15/98.68	89.81/98.16	88.93/98.16

Table S2e. Nucleotide and protein homology of PA gene of ten strains.

Strain name	Sequence identities with some strains (nt/aa%)								
	G152_2019	G155_2019	G188_2019	G630_2019	H159_2020	34_2020	140_2020	144_2020	151_2020
G152_2019	-	89.73/98.05	90.38/98.47	90.38/99.02	90.61/98.61	93.35/98.33	90.52/98.19	90.66/98.61	90.75/98.61
G155_2019	89.73/98.05	-	91.68/98.19	91.63/98.74	91.59/98.61	89.68/97.49	91.12/98.19	91.26/98.61	91.35/98.61
G188_2019	90.38/98.47	91.68/98.19	-	94.33/99.16	95.82/99.30	90.70/98.47	95.54/98.88	95.68/99.30	95.86/99.30
G630_2019	90.38/99.02	91.63/98.74	94.33/99.16	-	94.24/99.30	90.66/98.47	93.96/98.88	94.10/99.30	94.19/99.30
H159_2020	90.61/98.61	91.59/98.61	95.82/99.30	94.24/99.30	-	90.84/98.61	99.44/99.58	99.58/100.00	99.77/100.00
34_2020	93.35/98.33	89.68/97.49	90.70/98.47	90.66/98.47	90.84/98.61	-	90.66/98.19	90.79/98.61	90.79/98.61
140_2020	90.52/98.19	91.12/98.19	95.54/98.88	93.96/98.88	99.44/99.58	90.66/98.19	-	99.86/99.58	99.49/99.58
144_2020	90.66/98.61	91.26/98.61	95.68/99.30	94.10/99.30	99.58/100.00	90.79/98.61	99.86/99.58	-	99.63/100.00
151_2020	90.75/98.61	91.35/98.61	95.86/99.30	94.19/99.30	99.77/100.00	90.79/98.61	99.49/99.58	99.63/100.00	-
157_2020	98.37/99.44	89.82/97.77	90.10/98.19	90.19/98.74	90.61/98.61	93.40/98.33	90.52/98.19	90.66/98.61	90.75/98.61

Table S2f. Nucleotide and protein homology of NP gene of ten strains.

Strain name	Sequence identities with some strains (nt/aa%)								
	G152_2019	G155_2019	G188_2019	G630_2019	H159_2020	34_2020	140_2020	144_2020	151_2020
G152_2019	-	95.59/98.80	92.12/98.60	92.38/98.80	91.12/98.60	91.85/98.80	91.92/99.00	91.12/98.60	91.18/98.60
G155_2019	95.59/98.80	-	91.78/97.80	92.05/98.40	91.32/98.20	91.58/98.00	91.78/98.60	91.32/98.20	91.38/98.20
G188_2019	92.12/98.60	91.78/97.80	-	96.53/99.00	95.86/98.80	99.20/99.80	96.66/98.80	95.86/98.80	95.93/98.80
G630_2019	92.38/98.80	92.05/98.40	96.53/99.00	-	95.86/99.00	96.33/99.20	97.13/99.40	95.86/99.00	95.93/99.00
H159_2020	91.12/98.60	91.32/98.20	95.86/98.80	95.86/99.00	-	95.86/99.00	98.60/99.60	100.00/100.00	99.93/100.00
34_2020	91.85/98.80	91.58/98.00	99.20/99.80	96.33/99.20	95.86/99.00	-	96.66/99.00	95.86/99.00	95.93/99.00
140_2020	91.92/99.00	91.78/98.60	96.66/98.80	97.13/99.40	98.60/99.60	96.66/99.00	-	98.60/99.60	98.66/99.60
144_2020	91.12/98.60	91.32/98.20	95.86/98.80	95.86/99.00	100.00/100.00	95.86/99.00	98.60/99.60	-	99.93/100.00
151_2020	91.18/98.60	91.38/98.20	95.93/98.80	95.93/99.00	99.93/100.00	95.93/99.00	98.66/99.60	99.93/100.00	-
157_2020	91.05/98.40	91.38/98.00	96.06/99.00	95.93/99.20	99.67/99.80	95.93/99.20	98.53/99.40	99.67/99.80	99.73/99.80

Table S2g. Nucleotide and protein homology of NS gene of ten strains.

Strain name	Sequence identities with some strains (nt/aa%)								
	G152_2019	G155_2019	G188_2019	G630_2019	H159_2020	34_2020	140_2020	144_2020	151_2020
G152_2019	-	98.24/97.54	93.07/91.90	94.36/93.31	93.18/90.49	93.54/91.90	93.30/91.20	93.30/91.20	93.54/93.31
G155_2019	98.24/97.54	-	93.89/92.96	94.95/94.01	94.01/91.55	94.36/92.96	94.12/92.25	94.12/92.25	94.59/94.37
G188_2019	93.07/91.90	93.89/92.96	-	94.01/92.61	97.88/97.89	98.59/99.30	98.24/98.59	98.24/98.59	92.95/90.49
G630_2019	94.36/93.31	94.95/94.01	94.01/92.61	-	93.89/91.20	94.48/92.61	94.24/91.90	94.24/91.90	93.18/92.25
H159_2020	93.18/90.49	94.01/91.55	97.88/97.89	93.89/91.20	-	99.06/97.89	99.65/99.30	99.65/99.30	93.89/90.49
34_2020	93.54/91.90	94.36/92.96	98.59/99.30	94.48/92.61	99.06/97.89	-	99.41/98.59	99.41/98.59	93.89/90.49
140_2020	93.30/91.20	94.12/92.25	98.24/98.59	94.24/91.90	99.65/99.30	99.41/98.59	-	100.00/100.00	94.01/91.20
144_2020	93.30/91.20	94.12/92.25	98.24/98.59	94.24/91.90	99.65/99.30	99.41/98.59	100.00/100.00	-	94.01/91.20
151_2020	93.54/93.31	94.59/94.37	92.95/90.49	93.18/92.25	93.89/90.49	93.89/90.49	94.01/91.20	94.01/91.20	-
157_2020	94.24/95.07	95.30/96.13	93.54/92.25	95.42/95.42	93.30/90.85	94.01/92.25	93.65/91.55	93.65/91.55	97.06/96.48

Table S2h. Nucleotide and protein homology of M gene of ten strains.

Strain name	Sequence identities with some strains (nt/aa%)								
	G152_2019	G155_2019	G188_2019	G630_2019	159_2020	34_2020	140_2020	144_2020	151_2020
G152_2019	-	95.11/97.87	96.33/98.78	95.42/97.26	96.44/99.09	95.42/98.78	96.44/99.09	96.54/99.39	96.13/97.87
G155_2019	95.11/97.87	-	96.64/97.87	97.45/98.17	96.74/98.17	96.33/98.48	96.74/98.17	96.84/98.48	96.23/96.95
G188_2019	96.33/98.78	96.64/97.87	-	97.15/97.26	98.88/99.09	97.25/98.78	98.88/99.09	98.98/99.39	98.37/97.87
G630_2019	95.42/97.26	97.45/98.17	97.15/97.26	-	97.05/97.56	96.64/97.26	97.05/97.56	97.15/97.87	96.54/96.34
159_2020	96.44/99.09	96.74/98.17	98.88/99.09	97.05/97.56	-	96.95/99.09	99.80/99.39	99.90/99.70	99.49/98.78
34_2020	95.42/98.78	96.33/98.48	97.25/98.78	96.64/97.26	96.95/99.09	-	96.95/99.09	97.05/99.39	96.44/97.87
140_2020	96.44/99.09	96.74/98.17	98.88/99.09	97.05/97.56	99.80/99.39	96.95/99.09	-	99.90/99.70	99.29/98.17
144_2020	96.54/99.39	96.84/98.48	98.98/99.39	97.15/97.87	99.90/99.70	97.05/99.39	99.90/99.70	-	99.39/98.48
151_2020	96.13/97.87	96.23/96.95	98.37/97.87	96.54/96.34	99.49/98.78	96.44/97.87	99.29/98.17	99.39/98.48	-
157_2020	96.54/99.39	96.84/98.48	98.98/99.39	97.15/97.87	99.90/99.70	97.05/99.39	99.90/99.70	100.00/100.00	99.39/98.48
