# Supplementary material for: A Characterization and an Evolutionary and a Pathogenicity Analysis of Reassortment H3N2 Avian Influenza Virus in South China in 2019–2020
Source: Viruses. 2022 Nov 21;14(11):2574. doi: 10.3390/v14112574 (PMC9692712; doi:10.3390/v14112574)
Supplement: Supplementary file 1 [file viruses-14-02574-s001.zip › Supplementary table S3.pdf]

Table S3. Similarity analysis of internal gene.

Strain name	Gene	Closest virus	Homology (%)
H34	HA	A/duck/Hunan/7/2015(H3N6)	96.47
	NA	A/duck/China/322D22/2018(H3N2)	98.02
	M	A/duck/Vietnam/LBM48/2011(H3N2)	98.07
	NP	A/chicken/Ganzhou/GZ157/2016(H3N2)	97.8
	NS	A/chicken/Ganzhou/GZ43/2016(H3N2)	98.2
	PA	A/duck/China/322D22/2018(H3N2)	98.03
	PB1	A/duck/China/322D22/2018(H3N2)	97.95
	PB2	A/duck/Guangxi/293D21/2017(H1N2)	98.85
H159	HA	A/duck/Hunan/7/2015(H3N6)	96.36
	NA	A/duck/Zhejiang/727042/2014(H6N2)	96.45
	M	A/duck/China/322D22/2018(H3N2)	99.42
	NP	A/duck/Guangdong/S4040/2011(H4N2)	100
	NS	A/chicken/Ganzhou/GZ43/2016(H3N2)	97.53
	PA	A/chicken/Ganzhou/GZ157/2016(H3N2)	97.81
	PB1	A/duck/Guangxi/293D21/2017(H1N2)	97.74
	PB2	A/duck/Guangxi/293D21/2017(H1N2)	97.78
G188	HA	A/duck/Hubei/ZYSYF18/2015(H3N6)	97.51
	NA	A/chicken/Ganzhou/GZ43/2016(H3N2)	98.08
	M	A/duck/China/322D22/2018(H3N2)	99.32
	NP	A/chicken/Ganzhou/GZ157/2016(H3N2)	97.93
	NS	A/chicken/Ganzhou/GZ43/2016(H3N2)	98.43
	PA	A/chicken/Ganzhou/GZ43/2016(H3N2)	97.54
	PB1	A/duck/Hubei/ZYSYF2/2015(H3N6)	98.3
	PB2	A/chicken/Guangxi/165C7/2014(H3N2)	97.31
G630	HA	A/duck/Hubei/ZYSYF18/2015(H3N6)	95.53
	NA	A/duck/Guangdong/8.30_DGCP036-C/2017(H6N2)	98.09
	M	A/chicken/Zhejiang/102622/2016(H10N8)	99.29
	NP	A/duck/Jiangxi/22215/2013(H7N3)	99.4
	NS	A/chicken/Zhejiang/51048/2015(H1N9)	98.47
	PA	A/chicken/Yuhuan/YH14/2016(H1N2)	97.90

	PB1	**A/chicken/Zhejiang/51048/2015(H1N9)**	97.5
	PB2	**A/duck/Yuhuan/YH45/2016(H1N2)**	97.86
H144	HA	**A/duck/Hunan/7/2015(H3N6)**	96.41
	NA	**A/duck/Zhejiang/727042/2014(H6N2)**	96.52
	M	**A/duck/China/322D22/2018(H3N2)**	99.51
	NP	**A/duck/Vietnam/HN5001/2018(H3N2)**	97.38
	NS	**A/chicken/Ganzhou/GZ43/2016(H3N2)**	97.87
	PA	**A/chicken/Ganzhou/GZ157/2016(H3N2)**	97.68
	PB1	**A/duck/Guangxi/293D21/2017(H1N2)**	97.78
	PB2	**A/duck/Guangxi/293D21/2017(H1N2)**	97.65
H140	HA	**A/duck/Hunan/7/2015(H3N6)**	96.41
	NA	**A/duck/Zhejiang/727042/2014(H6N2)**	96.45
	M	**A/duck/China/322D22/2018(H3N2)**	99.42
	NP	**A/duck/Guangdong/S4040/2011(H4N2)**	98.80
	NS	**A/chicken/Ganzhou/GZ43/2016(H3N2)**	97.87
	PA	**A/chicken/Ganzhou/GZ157/2016(H3N2)**	97.54
	PB1	**A/duck/Guangxi/293D21/2017(H1N2)**	97.78
	PB2	**A/duck/Guangxi/293D21/2017(H1N2)**	98.50
H151	HA	**A/duck/Hunan/7/2015(H3N6)**	96.47
	NA	**A/duck/Guangxi/293D21/2017(H1N2)**	96.19
	M	**A/duck/China/322D22/2018(H3N2)**	98.93
	NP	**A/duck/Guangdong/S4040/2011(H4N2)**	97.46
	NS	**A/environment/Bangladesh/42635/2020(H10N7)**	96.99
	PA	**A/chicken/Ganzhou/GZ157/2016(H3N2)**	97.95
	PB1	**A/Mallard (Anas platyrhynchos)/South Korea/KNU2019-33/2019(H7N7)**	99.12

	PB2	**A/chicken/Shandong/10.23_TAWL012-O/2018(H9N2)**	92.15
H157	HA	**A/duck/Hunan/7/2015(H3N6)**	97.18
	NA	**A/duck/Zhejiang/727042/2014(H6N2)**	96.45
	M	**A/duck/China/322D22/2018(H3N2)**	99.51
	NP	**A/duck/Guangdong/S4040/2011(H4N2)**	97.46
	NS	**A/environment/Bangladesh/42635/2020(H10N7)**	99.19
	PA	**A/Anas platyrhynchos/Belgium/11958/2018(H1N1)**	98.87
	PB1	**A/duck/Guangxi/293D21/2017(H1N2)**	97.82
	PB2	**A/Mallard (Anas platyrhynchos)/South Korea/KNU2019-61/2019(H4N6)**	93.67
G152	HA	**A/chicken/Guangxi/165C7/2014(H3N2)**	95.88
	NA	**A/chicken/Guangxi/165C7/2014(H3N2)**	96.03
	M	**A/duck/Mongolia/619/2019(H3N6)**	99.7
	NP	**A/duck/Zhejiang/422/2013(H4N6)**	97.13
	NS	**A/mallard/Xuyi/14/2015(H3N8)**	97.30
	PA	**A/duck/Bangladesh/38827/2019(H11N3)**	99.13
	PB1	**A/duck/Guangxi/293D21/2017(H1N2)**	98.42
	PB2	**A/duck/Zhejiang/6D7/2013(H3N2)**	94.74
G155	HA	**A/chicken/Guangxi/165C7/2014(H3N2)**	96.65
	NA	**A/duck/Guangdong/S1469/2010(H4N2)**	94.47
	M	**A/duck/Yuhuan/YH45/2016(H1N2)**	98.15
	NP	**A/chicken/Guangxi/165C7/2014(H3N2)**	96.1
	NS	**A/mallard/Xuyi/14/2015(H3N8)**	97.87
	PA	**A/duck/Japan/AQ-HE103/2015(H1N2)**	95.84
	PB1	**A/duck/Japan/AQ-HE103/2015(H1N2)**	96.12
	PB2	**A/chicken/Guangxi/165C7/2014(H3N2)**	95.31
