# Supplementary material for: A Characterization and an Evolutionary and a Pathogenicity Analysis of Reassortment H3N2 Avian Influenza Virus in South China in 2019–2020
Source: Viruses. 2022 Nov 21;14(11):2574. doi: 10.3390/v14112574 (PMC9692712; doi:10.3390/v14112574)
Supplement: Supplementary file 1 [file viruses-14-02574-s001.zip › Supplementary table S4.pdf]

Table S4 The Substitution Rate of the eight segments.

Gene	Substitution Rate and 95% HPD (10^{-3} subs/site/year)			ESS
	Mean	Lower	Upper	
PB2	4.9936	4.0646	5.9315	1407.2
PB1	2.7645	2.266	3.2758	1490.3
PA	2.724	2.3898	3.0545	2357.4
HA	3.447	2.8667	4.0875	456.9
NP	2.6903	2.2769	3.1301	1483.2
NA	4.2913	3.7177	4.8743	1167.1
MP	2.1103	1.6106	2.6038	7347
NS	2.5366	1.8624	3.2288	1321.6
