# Supplementary material for: A Characterization and an Evolutionary and a Pathogenicity Analysis of Reassortment H3N2 Avian Influenza Virus in South China in 2019–2020
Source: Viruses. 2022 Nov 21;14(11):2574. doi: 10.3390/v14112574 (PMC9692712; doi:10.3390/v14112574)
Supplement: Supplementary file 1 [file viruses-14-02574-s001.zip › Supplementary table S5.pdf]

Table S5. The Most recent common ancestor of eight segments from all isolates.

segment	R^2	Best-fit model	strain	Most recent common ancestor	Posterior
PB2	0.6217	GTR+F+G4	H159_2020	A_duck_China_322D22_2018_H3N2	0.8888
			H144_2020		
			H140_2020		
			H34_2020		
			H157_2020		
			H151_2020		
			G155_2019	A_duck_Zhejiang_6D4_2013_H3N2	0.859
			G152_2019		
			G630_2019	A_chicken_Yuhuan_YH14_2016_H1N2	0.5352
			G188_2019	A_chicken_Guangxi_165C7_2014_H3N2	0.7575
PB1	0.6244	GTR+F+G4	H159_2020	A_duck_Guangxi_293D21_2017_H1N2	1
			H144_2020		
			H157_2020		
			H140_2020		
			H34_2020		
			G152_2019		
			G155_2019	A_duck_Guangdong_04_22_DGCP064_P_2015_Mixed_H3N2	0.9479
			G188_2019	A_duck_Hubei_ZYSYF2_2015_H3N6	1
			G630_2019	A_chicken_Zhejiang_51043_2015_H1N9	0.9956
			H151_2020	A_duck_Mongolia_837_2015_H1N1	1
PA	0.6642	GTR+F+G4	H151_2020	A_chicken_Ganzhou_GZ157_2016_H3N2	1
			H144_2020		
			H159_2020		
			H140_2020		
			G188_2019	A_chicken_Ganzhou_GZ43_2016_H3N2	0.9996
			G155_2019	A_duck_Japan_AQ_HE103_2015_H1N2	1
			G630_2019	A_chicken_Zhejiang_51048_2015_H1N9	0.9999
			H34_2020	A_duck_China_322D22_2018_H3N2	1
			G152_2019	A_common_teal_Shanghai_NH110923_2019_H1N1	1

			H157_2020	A_duck_Bangladesh_19D668_2016_H2N3	0.9998
HA	0.5091	GTR+F+G4	G188	A_duck_Hunan_7_2015_H3N6	0.9911
			H34_2020	A_duck_Hunan_161_2015_H3N6	0.9357
			H157_2020		
			H140_2020		
			H159_2020		
			H144_2020		
			H151_2020		
			G630_2019		
			G155_2019	A_duck_Guangdong_04_16_SZLGWL012_2015_Mixed_H3N6	0.9996
			G152_2019		
NP	0.5195	GTR+F+G4	H144_2020	A_duck_Hunan_7_2015_H3N6	0.9644
			H159_2020		
			H151_2020		
			H140_2020		
			H157_2020		
			H34_2020	A_duck_Guangxi_04_10_JX050_2015_Mixed_H3N2	0.6315
			G188_2019		
			G630_2019	A_duck_Jiangxi_22215_2013_H7N3	0.997
			G152_2019	A_duck_Zhejiang_422_2013_H4N6	0.998
			G155_2019	A_duck_Jiangxi_01_14_NCJD060_P_2015_Mixed_H3N6	0.4717
NA	0.5277	GTR+F+G4	H159_2020	A_duck_Guangxi_293D21_2017_H1N2	1
			H157_2020		
			H140_2020		
			H144_2020		
			H151_2020		
			H34_2020	A_duck_Guangdong_F352_2018_H3N2	1
			G188_2019	A_duck_Jiangshu_YZ916_2016_H3N2	1
			G152_2019	A_duck_Fujian_05_07_FZHD5_P_2015_Mixed_H6N2	0.9337
			G155_2019		
			G630_2019	A_duck_Guangdong_8_30_DGCP036_C_2017_H6N2	1
MP	0.43	TN93+all	G188_2019	A_duck_China_322D22_2018_H3N2	0.9997
			H151_2020		
			H140_2020		
			H144_2020		
			H157_2020		
			H159_2020		
			G630_2019	A_chicken_Zhejiang_102622_2016_	0.9904

		equal +G4		10_26_H10N8	
			G155_2019	A_duck_Guangdong_04_22_DGCP075_P_2015_Mixed_H3N2	0.9583
			H34_2020	A_duck_Jiangshu_YZ916_2016_H3N2	0.5736
			G152_2019	A_mallard_Sanjiang_90_2006_H3N8	0.9984
NS	0.563	GTR+F+G4	H157_2020	A_common_teal_Shanghai_NH110923_2019_H1N1	0.962
			H151_2020		
			G630_2019	A_chicken_Zhejiang_51043_2015_H1N9	0.9899
			H140_2020	A_chicken_Ganzhou_GZ157_2016_H3N2	0.9952
			H159_2020		
			H144_2020		
			H34_2020		
			G188_2019		
			G152_2019	A_chicken_Guangxi_165C7_2014_H3N2	0.6475
			G155_2019		
