# Supplementary material for: A Characterization and an Evolutionary and a Pathogenicity Analysis of Reassortment H3N2 Avian Influenza Virus in South China in 2019–2020
Source: Viruses. 2022 Nov 21;14(11):2574. doi: 10.3390/v14112574 (PMC9692712; doi:10.3390/v14112574)
Supplement: Supplementary file 1 [file viruses-14-02574-s001.zip › Supplementary table S6.pdf]

Table S6. Molecular character of the HA gene.

strains	Q226L	G228S	158-160	324-332	98	138	153	183	190
	antigen-binding site	Antigen binding site	glycosylation site	Cleavage site	Key receptor binding sites				
G152	Q	G	GSS	PEKQTR ↑ GLF	Y	A	W	H	E
G155	Q	G	GNS	PEKQTR ↑ GLF	Y	A	W	H	E
G188	Q	G	GNS	PEKQTR ↑ GLF	Y	A	W	H	E
G630	Q	G	GNS	PEKQTR ↑ GLF	Y	A	W	H	E
H34	Q	G	GNS	PEKQTR ↑ GLF	Y	A	W	H	E
H140	Q	G	GNS	PEKQTR ↑ GLF	Y	A	W	H	E
H144	Q	G	GNS	PEKQTR ↑ GLF	Y	A	W	H	E
H151	Q	G	GNS	PEKQTR ↑ GLF	Y	A	W	H	E
H157	Q	G	GNS	PEKQTR ↑ GLF	Y	A	W	H	E
H159	Q	G	GNS	PEKQTR ↑ GLF	Y	A	W	H	E

Segment	Position	G152	G155	G188	G630	H34	H140	H144	H151	H157	H159	Mutation	Function
PB2	D253	D	D	D	D	D	D	D	D	D	D	D→N	Contributes to the Pathogenesis of the Virus in Mammalian Hosts(Jinfeng *et al.*, 2018)
	292	I	I	I	I	I	I	I	I	I	I	V→I	abolished the transmission of H7N9(Kong *et al.*, 2019)
	389	R	R	R	R	R	R	R	K	R	R	R→K	higher adaptability of AIVs in avians and mammals(Li *et al.*, 2022)
	588	A	A	A	A	A	S	S	A	S	S	A→S	promotes the mammalian adaptation (Xiao *et al.*, 2016)
	598	T	T	T	T	T	T	T	T	T	T	T→M	higher adaptability of AIVs in avians and mammals(Li *et al.*, 2022)
	627	E	E	E	E	E	E	E	E	E	E	K→E	abolished the transmission of H7N9(Kong *et al.*, 2019)
	701	D	D	D	D	D	D	D	D	D	D	D→N	Promote Adaptation of an Influenza H5N1 Virus to a Mammalian Host(Czudai-Matwich *et al.*, 2014)
	648	L	L	L	L	L	L	L	I	L	I	L→V	play critical roles in mammalian adaptation(Xiao *et al.*, 2016)
	676	T	T	T	T	T	T	T	T	T	T	T→M	play critical roles in mammalian adaptation(Xiao *et al.*, 2016)
	714	S	S	S	S	S	S	S	S	S	S	S→R	Promote Adaptation of an Influenza H5N1 Virus to a Mammalian Host(Czudai-Matwich *et al.*, 2014)

PB1	269	S	S	S	S	S	S	S	S	S	S	F→S	increase the replication or virulence of avian influenza viruses in mammalian hosts(Guan *et al.*, 2019)
	436	Y	Y	Y	Y	Y	Y	Y	Y	Y	Y	Y→H	Reduce the virus fit(Hulse-Post *et al.*, 2007)
	577	K	K	K	K	K	K	K	K	K	K	K→E	Increases Polymerase Activity and Pathogenicity in Mice(Kamiki *et al.*, 2018)
	G622	G	G	G	G	G	G	G	G	G	G	G→D	attenuating H5N1 virus virulence in mice(Feng *et al.*, 2016)
	677	T	T	T	T	T	T	T	T	T	T	M→T	increase the replication or virulence of avian influenza viruses in mammalian hosts(Guan *et al.*, 2019)
	26	E	E	E	E	E	E	E	E	E	E	E→K	attenuates virus replication and pathogenicity(Clements *et al.*, 2020)
	224	S	S	S	S	S	S	S	S	S	S	S→P	increased the replication of the virus(Song *et al.*, 2011)
PA	343	A	A	A	A	A	A	A	A	A	A	A→S	increased viral polymerase activity and mouse virulence(Zhong *et al.*, 2018)
	347	D	D	D	D	D	D	D	D	D	D	E→D	increased viral polymerase activity and mouse virulence(Zhong *et al.*, 2018)
	356	K	K	K	K	K	K	K	K	K	K	K→R	Increases Mammalian Replication and Pathogenicity(Xu *et al.*, 2016)
	383	D	D	D	D	D	D	D	D	D	D	N→D	increased the polymerase activity(Song *et al.*, 2011)

	515	T	T	T	T	T	T	T	T	T	T	T→A	converted a lethal virus to a nonlethal virus in ducks(Song *et al.*, 2011)
	672	L	L	L	L	L	L	L	L	L	L	F→L	important role for airborne transmissibility among chickens(Zhong *et al.*, 2014)
NP	286	A	A	A	A	A	A	A	A	A	A	A→V	Attenuate H7N9 Viruses in Mice(Ma *et al.*, 2020)
	357	Q	Q	Q	Q	Q	Q	Q	Q	Q	Q	Q→K	Determines the virulence phenotype in mice(Zhu *et al.*, 2019)
	437	T	T	T	T	T	T	T	T	T	T	T→M	Attenuate H7N9 Viruses in Mice(Ma *et al.*, 2020)
NA	274	H	H	H	H	H	H	H	H	H	H	H→Y	conferring resistance to oseltamivir(Weinstock *et al.*, 2003)
	294	N	N	N	N	N	N	N	N	N	N	N→S	confer resistance to neuraminidase inhibitors(Kiso *et al.*, 2004)
MP	M1-30	D	D	D	D	D	D	D	D	D	D	N→D	increase the replication or virulence in mammalian(Shufang *et al.*, 2008)
	M1-156	D	D	D	D	D	D	D	D	D	D	D→E	abolished the transmission of H7N9(Kong *et al.*, 2019)
	M1-215	A	A	A	A	A	A	A	A	A	A	T→A	increase the replication or virulence in mammalian(Shufang *et al.*, 2008)
	M2-31	S	S	N	S	S	S	S	S	S	S	S→N/D	increase the resistance to adamantine(He *et al.*, 2021)

NS	42	S	S	S	S	S	S	S	S	S	S	S→P	increased virulence in mice(Jiao *et al.*, 2008)

References:

CLEMENTS, A. L., SEALY, J. E., PEACOCK, T. P., SADEYEN, J., HUSSAIN, S., LYCETT, S. J., SHELTON, H., DIGARD, P. & IQBAL, M. (2020), "Contribution of segment 3 to the acquisition of virulence in contemporary H9N2 avian influenza viruses.", *Journal of virology,* Vol. 94 No. 20, pp.

CZUDAI-MATWICH, V., OTTE, A., MATROSOVICH, M., GABRIEL, G. & KLENK, H. D. (2014), "PB2 mutations D701N and S714R promote adaptation of an influenza H5N1 virus to a mammalian host", *J Virol,* Vol. 88 No. 16, pp. 8735-42.

FENG, X., WANG, Z., SHI, J., DENG, G., KONG, H., TAO, S., LI, C., LIU, L., GUAN, Y. & CHEN, H. (2016), "Glycine at Position 622 in PB1 Contributes to the Virulence of H5N1 Avian Influenza Virus in Mice.", *Journal of virology,* Vol. 90 No. 4, pp.

GUAN, L., SHI, J., KONG, X., MA, S., ZHANG, Y., YIN, X., HE, X., LIU, L., SUZUKI, Y., LI, C., DENG, G. & CHEN, H. (2019), "H3N2 avian influenza viruses detected in live poultry markets in China bind to human-type receptors and transmit in guinea pigs and ferrets", *Emerg Microbes Infect,* Vol. 8 No. 1, pp. 1280-1290.

HE, W., ZHANG, W., YAN, H., XU, H., XIE, Y., WU, Q., WANG, C. & DONG, G. (2021), "Distribution and evolution of H1N1 influenza A viruses with adamantanes - resistant mutations worldwide from 1918 to 2019", *Journal of Medical Virology,* Vol. 93 No. 6, pp. 3473-3483.

HULSE-POST, D. J., FRANKS, J., BOYD, K., SALOMON, R., HOFFMANN, E., YEN, H. L., WEBBY, R. J., WALKER, D., NGUYEN, T. D. & WEBSTER, R. G. (2007), "Molecular changes in the polymerase genes (PA and PB1) associated with high pathogenicity of H5N1 influenza virus in mallard ducks", *J Virol,* Vol. 81 No. 16, pp. 8515-24.

JIAO, P., TIAN, G., LI, Y., DENG, G., JIANG, Y., LIU, C., LIU, W., BU, Z., KAWAOKA, Y. & CHEN, H. (2008), "A single-amino-acid substitution in the NS1 protein changes the pathogenicity of H5N1 avian influenza viruses in mice", *J Virol,* Vol. 82 No. 3, pp. 1146-54.

JINFENG, Z., RONG, S., XIAOYUN, J., HONGLIANG, A., RONBING, J. & CHRIS, K. P. M. (2018), "The D253N Mutation in the Polymerase Basic 2 Gene in Avian Influenza(H9N2) Virus Contributes to the Pathogenesis of the Virus in Mammalian Hosts", *Virologica Sinica,* Vol. 33 No. 06, pp. 531-537.

KAMIKI, H., MATSUGO, H., KOBAYASHI, T., ISHIDA, H., TAKENAKA-UEMA, A., MURAKAMI, S. & HORIMOTO, T. (2018), "A PB1-K577E Mutation in H9N2 Influenza Virus Increases Polymerase Activity and Pathogenicity in Mice", *Viruses,* Vol. 10 No. 11, pp.

KISO, M., MITAMURA, K., SAKAI-TAGAWA, Y., SHIRAISHI, K., KAWAKAMI, C., KIMURA, K., HAYDEN, F. G., SUGAYA, N. & KAWAOKA, Y. (2004), "Resistant

influenza A viruses in children treated with oseltamivir: descriptive study", *Lancet,* Vol. 364 No. 9436, pp. 759-65.
KONG, H., MA, S., WANG, J., GU, C., WANG, Z., SHI, J., DENG, G., GUAN, Y. & CHEN, H. (2019), "Identification of Key Amino Acids in the PB2 and M1 Proteins of H7N9 Influenza Virus That Affect Its Transmission in Guinea Pigs", *J Virol,* Vol. 94 No. 1, pp.
LI, B., SU, G., XIAO, C., ZHANG, J., LI, H., SUN, N., LAO, G., YU, Y., REN, X., QI, W., WANG, X. & LIAO, M. (2022), "The PB2 co-adaptation of H10N8 avian influenza virus increases the pathogenicity to chickens and mice", *Transbound Emerg Dis,* Vol. 69 No. 4, pp. 1794-1803.
MA, S., ZHANG, B., SHI, J., YIN, X., WANG, G., CUI, P., LIU, L., DENG, G., JIANG, Y., LI, C., CHEN, H. & PARRISH, C. R. (2020), "Amino Acid Mutations A286V and T437M in the Nucleoprotein Attenuate H7N9 Viruses in Mice", *Journal of virology,* Vol. 94 No. 2, pp.
SHUFANG, F., GUOHUA, D., JIASHENG, S., GUOBIN, T., YONGBING, S., YONGPING, J., YUNTAO, G., ZHIGAO, B., YOSHIHIRO, K. & HUALAN, C. (2008), "Two amino acid residues in the matrix protein M1 contribute to the virulence difference of H5N1 avian influenza viruses in mice", *Virology,* Vol. 384 No. 1, pp.
SONG, J., FENG, H., XU, J., ZHAO, D., SHI, J., LI, Y., DENG, G., JIANG, Y., LI, X., ZHU, P., GUAN, Y., BU, Z., KAWAOKA, Y. & CHEN, H. (2011), "The PA protein directly contributes to the virulence of H5N1 avian influenza viruses in domestic ducks.", *Journal of virology,* Vol. 85 No. 5, pp.
WEINSTOCK, D. M., GUBAREVA, L. V. & ZUCCOTTI, G. (2003), "Prolonged shedding of multidrug-resistant influenza A virus in an immunocompromised patient.", *The New England journal of medicine,* Vol. 348 No. 9, pp.
XIAO, C., MA, W., SUN, N., HUANG, L., LI, Y., ZENG, Z., WEN, Y., ZHANG, Z., LI, H., LI, Q., YU, Y., ZHENG, Y., LIU, S., HU, P., ZHANG, X., NING, Z., QI, W. & LIAO, M. (2016), "PB2-588 V promotes the mammalian adaptation of H10N8, H7N9 and H9N2 avian influenza viruses", *Sci Rep,* Vol. 619474.
XU, G., ZHANG, X., GAO, W., WANG, C., WANG, J., SUN, H., SUN, Y., GUO, L., ZHANG, R., CHANG, K. C., LIU, J. & PU, J. (2016), "Prevailing PA Mutation K356R in Avian Influenza H9N2 Virus Increases Mammalian Replication and Pathogenicity", *J Virol,* Vol. 90 No. 18, pp. 8105-14.
ZHONG, G., Le MQ, LOPES, T., HALFMANN, P., HATTA, M., FAN, S., NEUMANN, G. & KAWAOKA, Y. (2018), "Mutations in the PA Protein of Avian H5N1 Influenza Viruses Affect Polymerase Activity and Mouse Virulence", *J Virol,* Vol. 92 No. 4, pp.
ZHONG, L., WANG, X., LI, Q., LIU, D., CHEN, H., ZHAO, M., GU, X., HE, L., LIU, X., GU, M., PENG, D. & LIU, X. (2014), "Molecular mechanism of the airborne transmissibility of H9N2 avian influenza A viruses in chickens", *J Virol,* Vol. 88 No. 17, pp. 9568-78.
ZHU, W., FENG, Z., CHEN, Y., YANG, L., LIU, J., LI, X., LIU, S., ZHOU, L., WEI, H., GAO, R., WANG, D. & SHU, Y. (2019), "Mammalian-adaptive mutation NP-Q357K in Eurasian H1N1 Swine Influenza viruses determines the virulence phenotype in mice", *Emerg Microbes Infect,* Vol. 8 No. 1, pp. 989-999.
