# Supplementary figures and images for: A Characterization and an Evolutionary and a Pathogenicity Analysis of Reassortment H3N2 Avian Influenza Virus in South China in 2019–2020
Source: Viruses. 2022 Nov 21;14(11):2574. doi: 10.3390/v14112574 (PMC9692712; doi:10.3390/v14112574)

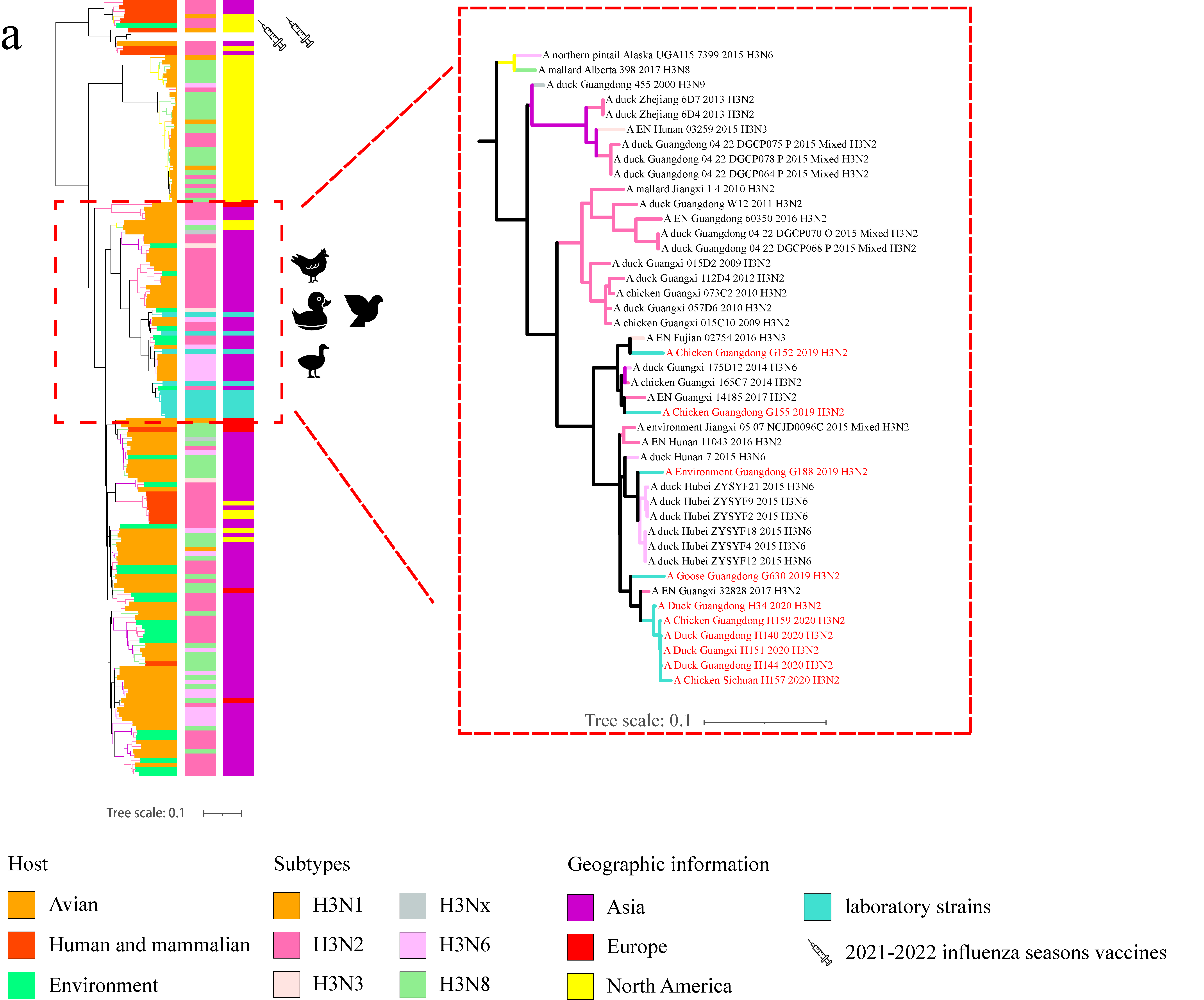

Supplement: Supplementary file 1 [file viruses-14-02574-s001.zip › Figure 1a.tif]

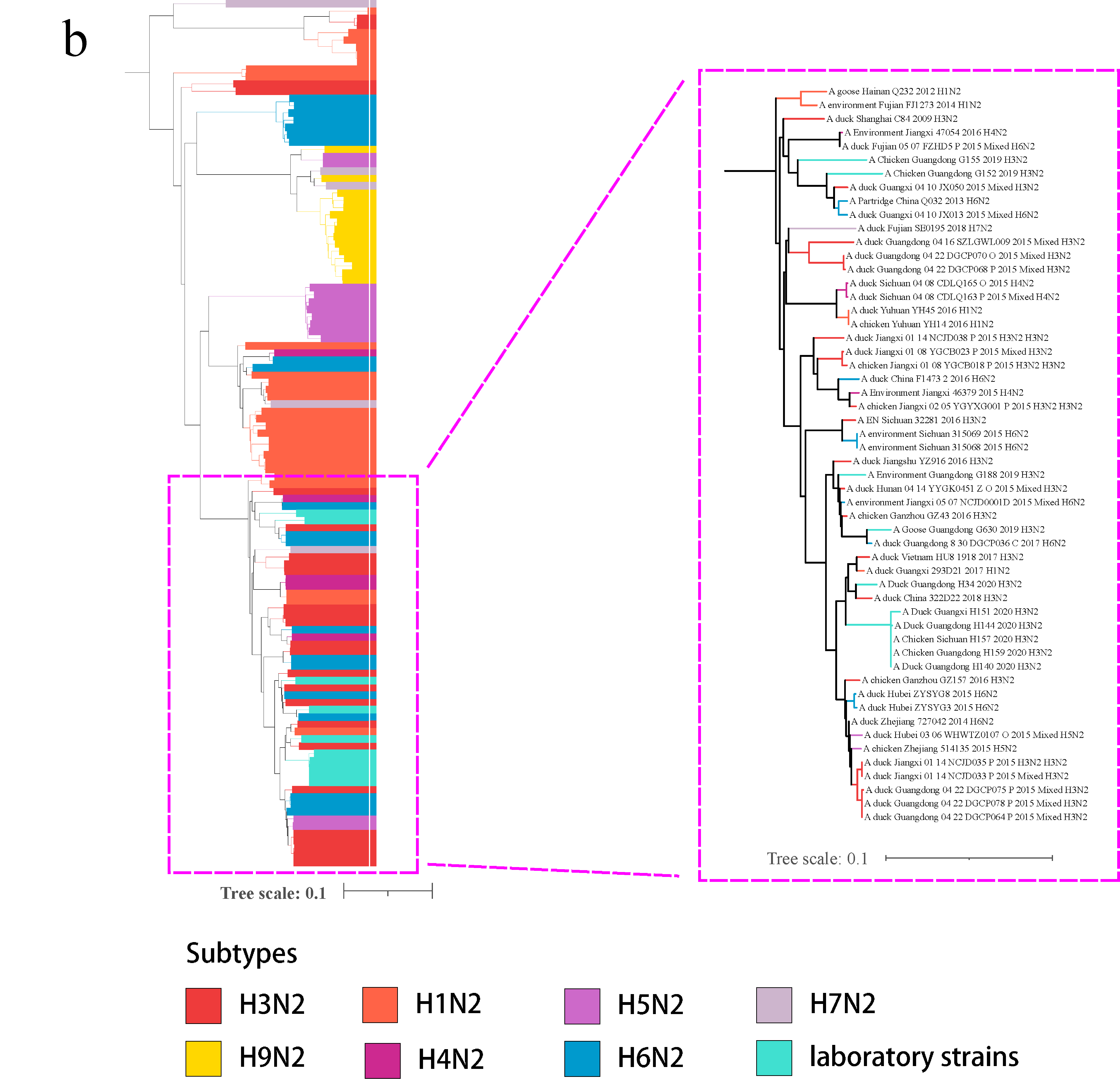

Supplement: Supplementary file 1 [file viruses-14-02574-s001.zip › Figure 1b.tif]

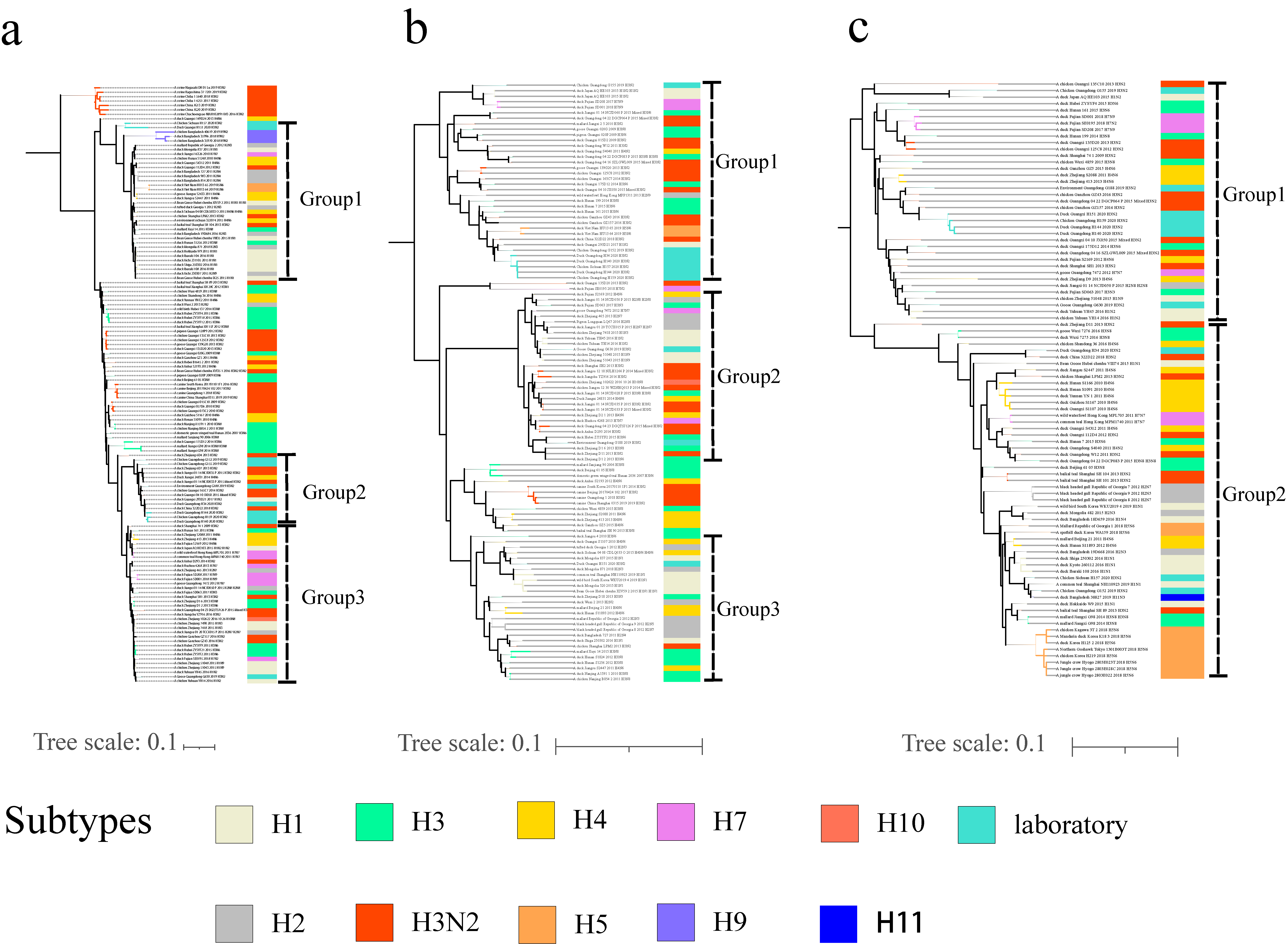

Supplement: Supplementary file 1 [file viruses-14-02574-s001.zip › Figure 2a-c.tif]

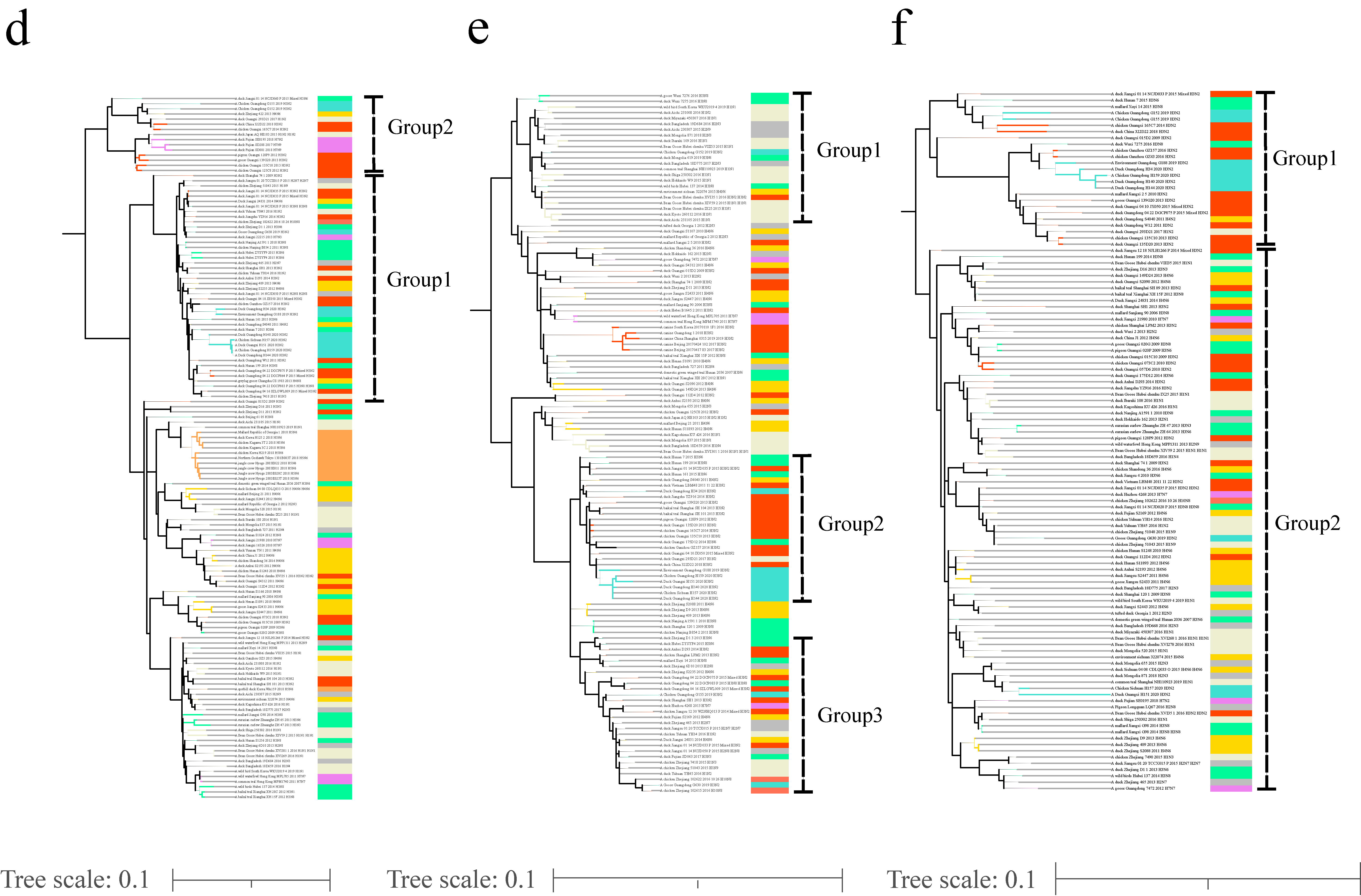

Supplement: Supplementary file 1 [file viruses-14-02574-s001.zip › Figure 2d-f.tif]

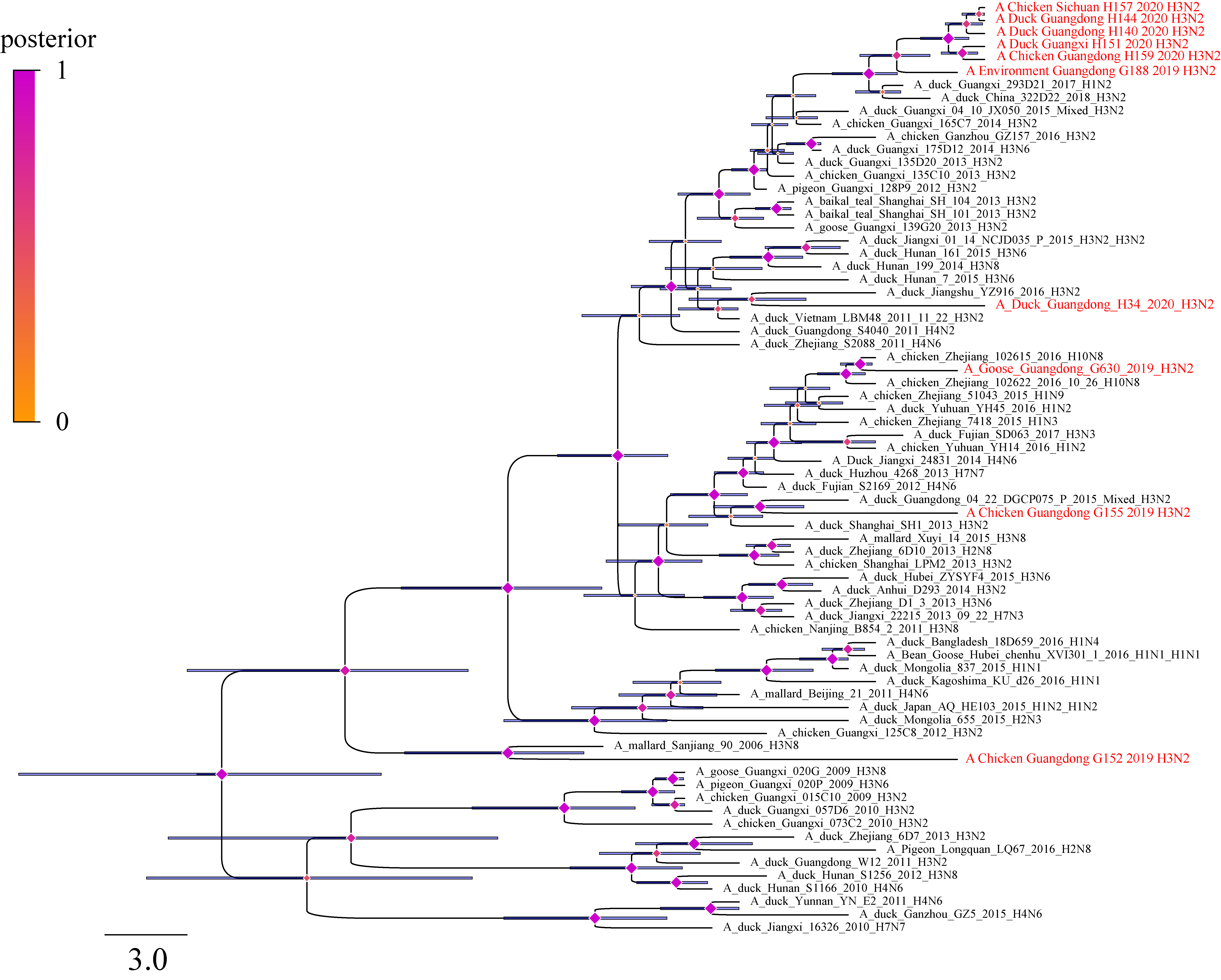

Supplement: Supplementary file 1 [file viruses-14-02574-s001.zip › Figure 3.tif]

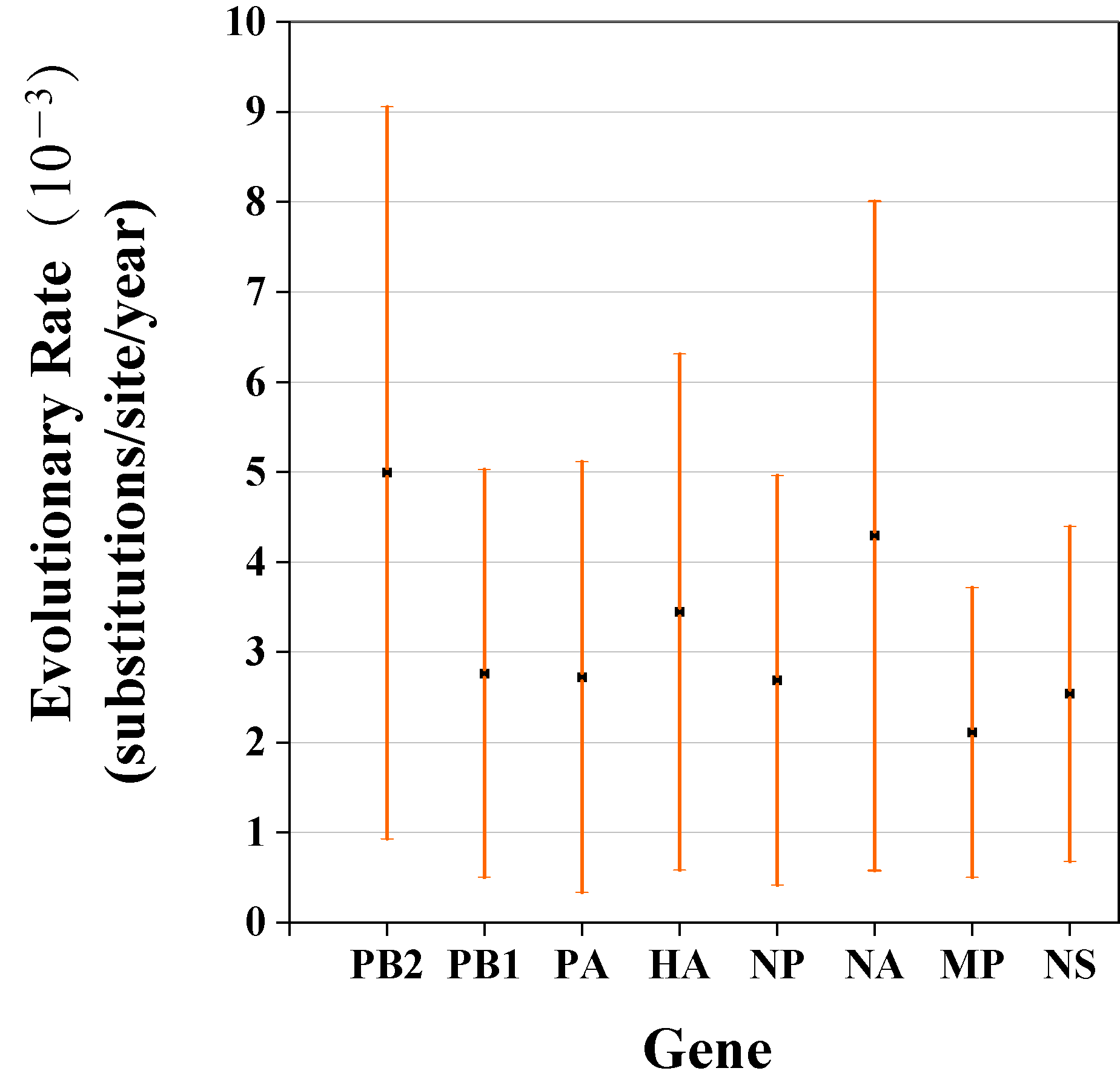

Supplement: Supplementary file 1 [file viruses-14-02574-s001.zip › Figure 4.tif]

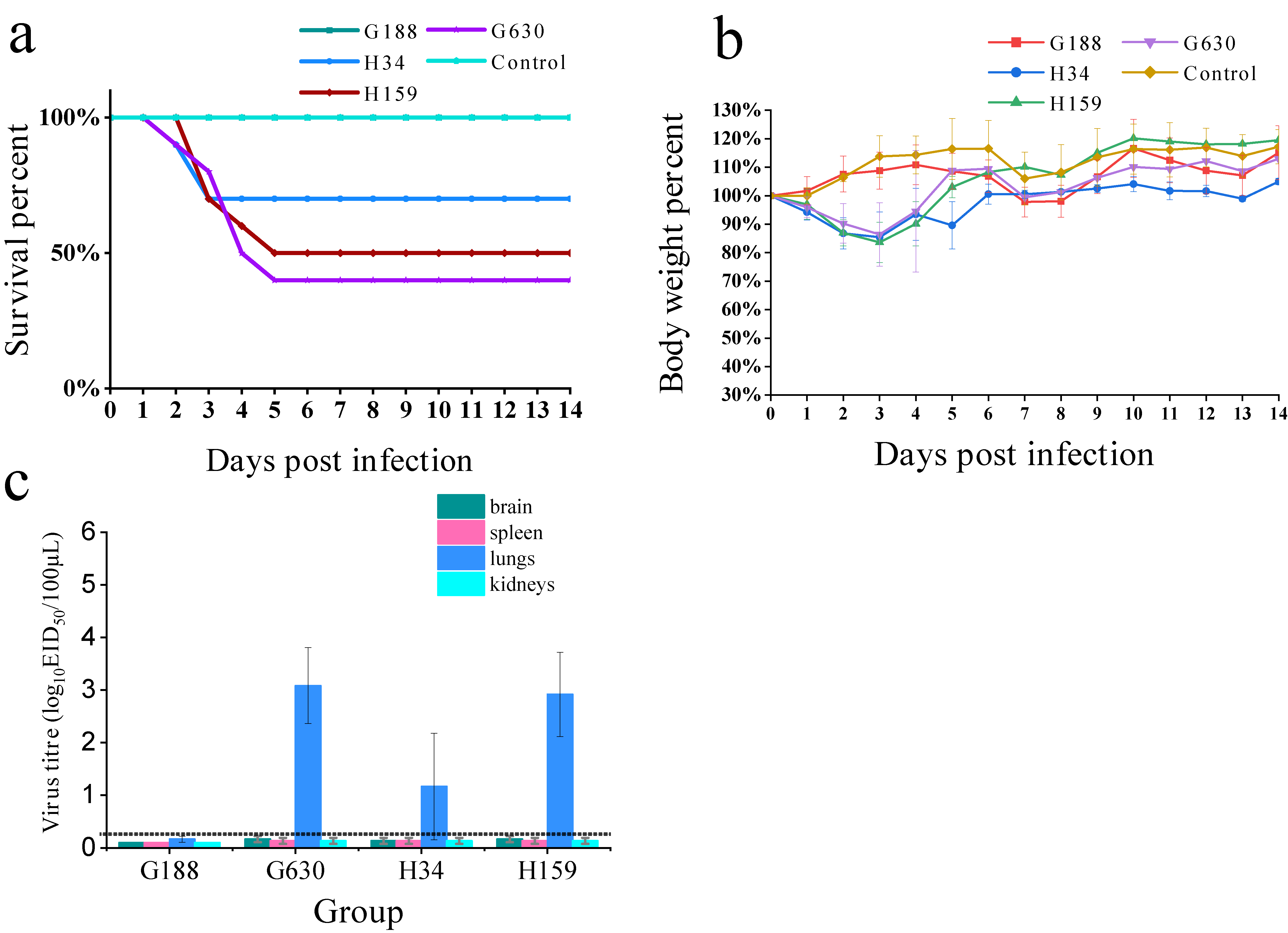

Supplement: Supplementary file 1 [file viruses-14-02574-s001.zip › Figure 5.tif]
